# ATPase-based implementation of enforced ATP wasting in *Saccharomyces cerevisiae* for improved ethanol production

**DOI:** 10.1186/s13068-020-01822-9

**Published:** 2020-11-09

**Authors:** Ahmed Zahoor, Katrin Messerschmidt, Simon Boecker, Steffen Klamt

**Affiliations:** Analysis and Redesign of Biological Networks, Max Planck Institute for Dynamics of Complex Technical Systems, Magdeburg, Germany

**Keywords:** Enforced ATP wasting, Ethanol, *Saccharomyces cerevisiae*, F_1_-ATPase, Metabolic engineering

## Abstract

**Background:**

Enforced ATP wasting has been recognized as a promising metabolic engineering strategy to enhance the microbial production of metabolites that are coupled to ATP generation. It also appears to be a suitable approach to improve production of ethanol by *Saccharomyces cerevisiae*. In the present study, we constructed different *S. cerevisiae* strains with heterologous expression of genes of the ATP-hydrolyzing F_1_-part of the ATPase enzyme to induce enforced ATP wasting and quantify the resulting effect on biomass and ethanol formation.

**Results:**

In contrast to genomic integration, we found that episomal expression of the αβγ subunits of the F_1_-ATPase genes of *Escherichia coli* in *S. cerevisiae* resulted in significantly increased ATPase activity, while neither genomic integration nor episomal expression of the β subunit from *Trichoderma reesei* could enhance ATPase activity. When grown in minimal medium under anaerobic growth-coupled conditions, the strains expressing *E. coli’s* F_1_-ATPase genes showed significantly improved ethanol yield (increase of 10% compared to the control strain). However, elevated product formation reduces biomass formation and, therefore, volumetric productivity. We demonstrate that this negative effect can be overcome under growth-decoupled (nitrogen-starved) operation with high and constant biomass concentration. Under these conditions, which mimic the second (production) phase of a two-stage fermentation process, the ATPase-expressing strains showed significant improvement in volumetric productivity (up to 111%) compared to the control strain.

**Conclusions:**

Our study shows that expression of genes of the F_1_-portion of *E. coli*’s ATPase induces ATPase activity in *S. cerevisiae* and can be a promising way to improve ethanol production*.* This ATP-wasting strategy can be easily applied to other metabolites of interest, whose formation is coupled to ATP generation.

## Background

Fossil fuels currently still serve as the major energy and chemical source worldwide. Reliance on them has several drawbacks including limited availability, uneven distribution around the world and the production of greenhouse gas emissions upon combustion. Consequently, there has been an increasing interest in development of biofuels as alternative to fossil fuels. This can be achieved, for example, at a biorefinery where multiple feedstock components can be used for the sustainable production of biofuels as well as of other industrially relevant chemicals [1].

Bioethanol is the biggest product of biotechnology, both in terms of economy and volume. Worldwide annual production is currently more than 100 billion L and it is expected to cross 134 billion L by 2024 [2]. *Saccharomyces cerevisiae* is the predominant organism used for bioethanol production and there is great interest in generating superior production strains [3, 4] since merely a 1% increase in ethanol yield can already save the industry millions of dollars annually [5]. Some characteristics of *S. cerevisiae* that make it an ideal host for industrial production are efficient anaerobic ethanol production, tolerance to ethanol and other stress factors such as low pH as well as its insensitivity to bacteriophage infection.

Several approaches have been implemented to generate improved ethanol-producing *S. cerevisiae* strains. Many of these strategies seek to either reduce production of ATP or to increase ATP turnover (enforced ATP wasting) (Table 1). The rationale behind this approach is that ethanol synthesis and ATP production are coupled in both directions (ethanol synthesis delivers ATP and (balanced) ATP synthesis implies synthesis of ethanol) and a loss of ATP must be counter-balanced by the cell with increased synthesis of ATP and thus of ethanol. One example of a strategy to reduce the amount of ATP produced is the effort to replace the Embden–Meyerhof–Parnas (EMP) pathway (yielding two moles of ATP per mole glucose) with the Entner–Doudoroff (ED) pathway (yielding only one mole ATP) [6]. This mimics the situation in *Zymomonas mobilis,* which employs the ED instead of the EMP pathway and, as a consequence of the reduced ATP yield, exhibits extremely high glucose uptake rates (60 mmol g^−1^ h^−1^) and 97% of the maximal ethanol yield [7]. The study [6] sought to implement the ED pathway in *S. cerevisiae* by expressing the corresponding genes from *E. coli*. However, one of the two enzymes required for this pathway (6-phosphogluconate dehydratase) failed to show activity despite several attempts to improve the iron–sulfur-cluster assembly which is critical for functioning of the enzyme [6].

**Table 1:** Overview of different ATP-wasting strategies applied in *S. cerevisiae*

	Benisch and Boles [6]	Semkiv et al*.* [14]	Semkiv et al*.* [19]	Navas et al*.* [13]	Basso et al*.* [8]	Jensen et al*.* [15, 21]	This study
ATP-wasting strategy	ED pathway	Futile cycle	Alkaline phosphatase	Futile cycle	Energy-dependent substrate uptake	F_1_-ATPase (β subunit)	F_1_-ATPase (different variants)
Use of F_1_-ATPase	No	No	No	No	No	Yes	Yes
Use of defined (minimal) medium	Yes	No	Yes	Yes	Yes	Yes	Yes
Detailed data on physiology of strains^a^	No	No	No	Yes	Yes	No	Yes
Anaerobic cultivation	No	No	No	No	Yes	No	Yes
ATP wasting for growth-coupled product formation	N.D.	Yes	Yes	Yes	Yes	Yes	Yes
ATP wasting for growth-decoupled product formation	N.D.	No	No	No	No	No	Yes

^a^Availability of comprehensive data on growth (biomass, growth rate), substrate utilization rates and production formation (titer, yield, and rates). N.D. not determined

Other studies showed that the use of alternative sugars (e.g., maltose or sucrose) or of alternative sugar transporters requiring more energy to transport the sugar into the cell may enhance the ethanol yield [5, 8–10]. Another approach to implement an ATP-wasting strategy is via the use of futile cycles, which are reaction cycles that result in net energy dissipation. In an *E. coli* lactate-producing strain, a futile cycle implemented by a combination of phosphoenolpyruvate synthase and pyruvate kinase, resulted in an increase of specific lactate productivity by almost 25% with a concomitant decrease in biomass yield of 26% [11, 12]. Similarly, in *S. cerevisiae*, two different futile cycles were tested for improvement of ethanol production. One consisted of a combined activity of phosphofructokinase and fructose-1,6-bisphosphatase resulting in an up to 9% improvement in ethanol titer. An alternative futile cycle based on the expression of pyruvate carboxylase and phosphoenolpyruvate carboxykinase genes also reported improved ethanol production per unit biomass in *S. cerevisiae* strains [13, 14].

Arguably, the simplest and most pragmatic method for enforced ATP wasting is the expression of a modified ATPase that can directly hydrolyze ATP. This approach was first implemented by Koebmann et al*.* [15, 16], who studied in *E. coli* the physiological effects of overexpressing the α, β and γ subunit genes of the cytosolic F_1_-portion of the ATPase catalyzing uncoupled ATP hydrolysis. Compared with other approaches, expression of such a single cytosolic ATP-hydrolyzing enzyme is less laborious and offers the additional benefit of not interfering with other intracellular metabolites as it directly hydrolyzes ATP to ADP. In prokaryotic hosts, expression of the cytosolic F_1_-ATPase has recently been shown to improve the production of metabolites that are coupled to ATP generation. For example, overexpressing genes of the native F_1_-ATPase in *E. coli* improved titer, yield and specific productivities of the fermentation products under growth-coupled conditions, as well as volumetric productivity during two-stage fermentation [17]. Similarly, expression of the F_1_-ATPase led to improved acetoin production in an engineered *Lactococcus lactis* strain [18].

The use of different enzymes that consume ATP has also been tested for improving ethanol production in *S. cerevisiae* [14, 19–21], including the use of the β subunit of the F_1_-portion of the ATPase from *Trichoderma reesei* [21]. However, these studies showed no or only minor enhancement of ethanol production or lack some critical information regarding the effect of such ATP wasting on the physiology of *S. cerevisiae*. Generally, we see the following drawbacks or gaps in earlier studies (Table 1): (a) the use of ATP-wasting mechanisms different from ATPase, which can interfere with central metabolism and may have unintended and unpredictable effects, (b) growth of strains in complex medium compromising yield calculations, (c) use of galactose as inducer (also used as carbon source by *S. cerevisiae*) again preventing unbiased yield and specific rate calculations, (d) comprehensive data on growth, substrate utilization rate and ethanol production (titer, yield, rates) are missing in most studies, and (e) growth-decoupled conditions were not considered.

Consequently, the aim of this study was to implement an ATP-wasting strategy in *S. cerevisiae* via the expression of genes of cytosolic ATPase enzyme(s) and to characterize the strains with respect to growth, ethanol production and substrate utilization. To this end, different variants of heterologous expression of genes of the F_1_-ATPase (or subunits of it) in *S. cerevisiae* were tested. As result, we found that episomal expression of the genes encoding the αβγ subunits of the F_1_-ATPase of *E. coli* results in significantly higher ATPase activity. Under anaerobic growth-coupled conditions, the ethanol titer and yield of these strains are significantly increased relatively to the control strain. The volumetric productivity decreased due to reduced biomass formation. In contrast, under growth-decoupled (nitrogen-starved) conditions with comparable biomass of the control and the ATPase strains, the volumetric productivity as well as specific production rates could be improved in comparison to the control strain.

## Results

### Heterologous expression of ATPase genes in *S. cerevisiae*

The objective of this study was to implement ATP wasting in *S. cerevisiae* by heterologous expression of genes of (suitable subunits of) an ATPase to improve ethanol production by the engineered strains. The F_o_F_1_-ATP synthase (or ATPase) is a ubiquitous enzyme that is made up of two distinct parts; the membrane bound F_o_ and the (water) soluble F_1_-part, both of which exist in the form of rotary motors. The cytoplasmic F_1_-part consists of five different subunits with the stoichiometry α_3_β_3_γδε whereby the minimal composition of the rotary motor is α_3_β_3_γ [22]. Whereas both α and β subunits can bind nucleotides, it is the β subunit where catalysis takes place [23]. Therefore, the first ATPase variant selected for expression in *S. cerevisiae* was the F_1_-ATPase β subunit from *T. reesei*, which, like *S. cerevisiae*, is a fungus. A previous patent application stated the use of *T. reesei* and provided preliminary evidence for its effect on ethanol production in *S. cerevisiae* [21]. In contrast to the *S. cerevisiae* β subunit, the *T. reesei* subunit lacks the mitochondrial signal peptide and hence can enable cytoplasmic ATPase activity.

Based on several positive reports [15–18, 24], the second and third variants of heterologous expression of F_1_-ATPase genes in *S. cerevisiae* are based on the *E. coli* F_1_-ATPase α, β and γ subunits. We first considered separate expression of each subunit gene under control of a strong constitutive promoter and strong terminator (see “Materials and methods”) as reported in the literature [25–27]. The yeast promoters and terminators are considerably longer (several hundred base pairs to more than 1 kb) compared to their prokaryotic counterparts. When making constructs with several genes, as in the case of *E. coli* F_1_-ATPase subunits, the repetitive use of these cis-acting elements makes the cloning process cumbersome and can exert extra drain on the machinery of the host cell. One of the strategies to decrease the construct size is to use the viral 2A sequences which are peptide sequences (usually up to 20 amino acids) that enable the expression of multiple genes from a polycistronic transcript and can thereby reduce the number of promoters needed. In recent years, several studies have reported the use of 2A sequences for the expression of multiple genes in yeasts [28–30]. As the third ATPase expression variant used in this study, the 2A sequences from Equine rhinitis B virus (ERBV-1) and Porcine teschovirus-1 (PTV) were used between the genes of the three α, β and γ subunits of the *E. coli* F_1_-ATPase. This reduced the construct size by more than 27% compared to the variant where each subunit has its own cis-acting elements.

There has been a noticeable increase in the availability of toolboxs for genetic engineering of *S. cerevisiae*. Highly efficient CRISPR-Cas9-based methods are available for marker-free insertions and deletions in *S. cerevisiae* genome [31–34]. Therefore, as a first attempt, we inserted either a single copy of the *T. reesei* F_1_-ATPase β subunit gene (*atp2*) or the three *E. coli* F_1_-ATPase subunits joined via viral 2A sequences into chromosome X of the prototrophic CEN.PK113-7D strain using the CRISPR-Cas9 marker-free kit [35] yielding strains 7D-*atp2* and 7D-*atpAGD* (2A), respectively. The parental (7D) and the transformed strains were grown in minimal medium, however, the transformed strains 7D-*atp2* and 7D-atp*AGD* (2A) did not show significant ATPase activity and no effect on biomass or ethanol production compared to the parental strain could be observed (data not shown). Consequently, a plasmid-based approach was followed for all three variants and a yeast episomal plasmid pRSII326 carrying 2µ circle replication origin and cis-acting STB (stability) locus was used for high copy expression of the different F_1_-ATPase genes [36]. The transformation of the uracil auxotrophic *S. cerevisiae* CEN.PK113-5D strain with the plasmid carrying *T. reesei* subunit resulted in the strain 5D-*atp2*. Likewise, transforming the plasmids with the α, β and γ subunit genes of *E. coli* with and without 2A sequences into *S. cerevisiae* CEN.PK113-5D yielded the strains 5D*-atpAGD* and 5D-*atpAGD* (2A), respectively (see “Materials and methods”).

The three plasmid-based ATPase strains were subsequently tested for functionality and for their effect on the production of ethanol under anaerobic growth-coupled and growth-decoupled (nitrogen-starved) conditions.

### Episomal expression of ***E. coli’s*** F_***1***_-ATPase genes induces high ATPase activity in ***S. cerevisiae***

We determined the ATPase activity in the control (5D (E.V.)) and in the three constructed strains with episomal expression of the F_1_-ATPase β subunit gene of *T. reesei* (5D-*atp2*) and of the *E. coli* F_1_-ATPase α_,_ β and γ subunit genes (5D-*atpAGD* and 5D-*atpAGD* (2A)), respectively. The strains were grown in minimal medium, pellets were made during exponential phase and cell lysate was used for determining the ATPase activity.

**Fig. 1:**
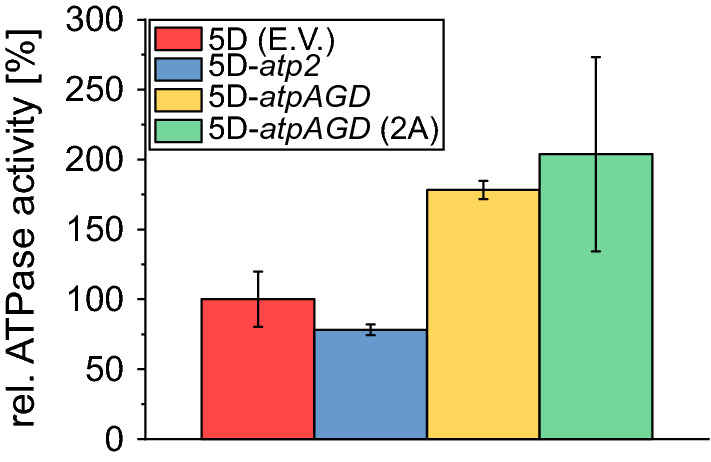
ATPase activities of cell lysates of the constructed strains. The *p* values for increased/decreased ATPase activity relative to the control strain are 0.1344 (5D-atp2), 0.0029 (5D-atpAGD) and 0.0677 (5D-atpAGD (2A)). The presented data are from triplicates and the error bars represent standard deviations

Compared to the control strain 5D (E.V.), the two strains expressing the three subunit genes of the *E. coli* F_1_-ATPase exhibit a clear increase in ATPase activity (Fig. 1) by 78% and 103%, respectively, which is in the range observed when overexpressing these genes directly in *E. coli* [17]. In contrast, the ATPase activity even decreased by 21% in the strain expressing the *T. reesei* F_1_-ATPase β subunit (5D-*atp2*), providing first evidence that this strain might not behave as intended.

### Effect of ATPase expression on growth-coupled ethanol production

**Fig. 2:**
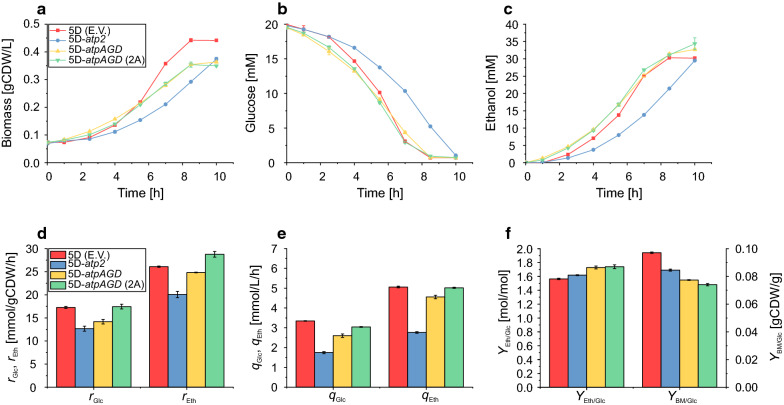
Biomass formation (**a**), glucose consumption (**b**), ethanol production (**c**), specific glucose uptake rate/ethanol productivity (**d**), volumetric glucose uptake rate/ethanol productivity after 7 h (**e**) and ethanol and biomass yield (**f**) of the constructed strains under growth-coupled conditions. The data presented are from triplicates and the error bars represent standard deviations. Specific and volumetric rate values are reported for the period where all strains showed exponential growth (2.5–7 h). Yields are reported for the entire period of cultivation (0–10 h)

After determining functional activity upon episomal expression of the heterologous ATPases, the effect on biomass and especially ethanol production was determined under anaerobic growth-coupled conditions. Again, the strains 5D-*atp2*, 5D-*atpAGD* and 5D-*atpAGD* (2A) were tested and compared with the control strain with empty vector. The strains were cultivated in minimal medium with 0.4% glucose and incubated anaerobically at 30 °C. Glucose uptake, biomass formation and production of ethanol and other byproducts were monitored and used to compute yields and specific consumption and production rates. To enable proper comparison, the specific rates were determined for identical time periods during exponential growth (from time point 2.5 h to 7 h for all strains; Fig. 2a–c; Table 2).

**Table 2:** Biomass, glucose uptake and production of ethanol, glycerol and acetate of the constructed *S. cerevisiae* strains under anaerobic conditions

	5D (E.V.)	5D-*atp2*	5D-*atpAGD*	5D-*atpAGD* (2A)
µ [h^−1^]	0.316 ± 0.005	0.201 ± 0.006 (*p* < 0.0001)	0.202 ± 0.003 (*p* < 0.0001)	0.235 ± 0.003 (*p* < 0.0001)
*Y*_BM/Glc_ [gCDW/g]	0.0971 ± 0.0007	0.0845 ± 0.0009 (*p* < 0.0001)	0.0774 ± 0.0003 (*p* < 0.0001)	0.0741 ± 0.0010 (*p* < 0.0001)
*r*_Glc_ [mmol/gCDW/h]	17.26 ± 0.27	12.66 ± 0.66 (*p* = 0.0004)	14.18 ± 0.60 (*p* = 0.0012)	17.44 ± 0.63 (*p* = 0.6712)
*q*_Glc_ [mmol/L/h]	3.35 ± 0.02	1.74 ± 0.07 (*p* < 0.0001)	2.60 ± 0.11 (*p* = 0.0003)	3.04 ± 0.03 (*p* < 0.0001)
*r*_Eth_ [mmol/gCDW/h]	26.09 ± 0.18	20.07 ± 0.84 (*p* = 0.0003)	24.82 ± 0.10 (*p* = 0.0004)	28.77 ± 0.76 (*p* = 0.0040)
*q*_Eth_ [mmol/L/h]	5.06 ± 0.05	2.76 ± 0.05 (*p* < 0.0001)	4.55 ± 0.10 (*p* = 0.0018)	5.02 ± 0.03 (*p* = 0.3346)
*Y*_Eth/Glc_ [mol/mol]	1.58 ± 0.01	1.62 ± 0.01 (*p* = 0.0042)	1.73 ± 0.03 (*p* = 0.0007)	1.74 ± 0.03 (*p* = 0.0014)
*r*_Gly_ [mmol/gCDW/h]	3.90 ± 0.22	2.89 ± 0.24 (*p* = 0.0059)	2.49 ± 0.16 (*p* = 0.0009)	3.39 ± 0.04 (*p* = 0.0172)
*Y*_Gly/Glc_ [mol/mol]	0.220 ± 0.003	0.201 ± 0.004 (*p* = 0.0040)	0.162 ± 0.006 (*p* = 0.0001)	0.185 ± 0.003 (*p* < 0.0001)
*r*_Ac_ [mmol/gCDW/h]	n.d.	n.d.	n.d.	n.d.
*Y*_Ac/Glc_ [mol/mol]	n.d.	n.d.	n.d.	n.d.
*r*_ATP-NGAM_ [mmol/gCDW/h]	0.30	3.66	8.33	9.59

Specific and volumetric rates are reported for the period where all strains showed exponential growth (2.5–7 h; see Fig. 2). The values of the yields are reported for the entire cultivation period (0–10 h). The data are from triplicate experiments and the error ranges represent standard deviations. *p* values for a two-sample *t* test are given with respect to the control strain 5D (E.V.) indicating whether a significant change has occurred

*Y*_BM/Glc_, biomass yield; *r*_Glc_, specific glucose uptake rate; *q*_Glc_*,* volumetric glucose uptake rate; *r*_Eth_, specific ethanol production rate; *q*_Eth_, volumetric ethanol productivity; *Y*_Eth/Glc_, ethanol yield; *r*_Gly_, specific glycerol production rate; *Y*_Gly/Glc_, glycerol yield; *r*_Ac_, specific acetate production rate; *Y*_Ac/Glc_, acetate yield; *r*_ATP-NGAM_, non-growth-associated ATP maintenance demand (estimated from flux balance calculations)

Despite the fact that the control strain already produces almost 80% of the maximal ethanol yield, heterologous expression of the F_1_-ATPases from *E. coli* significantly improved ethanol yield by more than 9% (5D-*atpAGD*) and 10% (5D-*atpAGD* (2A)), respectively, while strain 5D-*atp2* showed only a slight improvement of 2.5% (Fig. 2, Table 2). The specific glucose uptake rate is reduced in 5D-*atp2* and 5D-*atpAGD,* but is almost identical in 5D-*atpAGD* (2A) enabling this strain to reach also a higher specific ethanol productivity (10% increase relative to control strain). Using simple flux balance calculations, we estimated the non-growth associated ATP maintenance demand (which includes the amount of ATP hydrolyzed by the F_1_-ATPase) in all four strains (Table 2). These values clearly correlate with the ethanol yield and reach the highest value (9.59 mmol/gCDW/h) for strain 5D-*atpAGD* (2A) compared to 0.30 mmol/gCDW/h in the control strain.

As expected, ATPase expression had a significant impact on the biomass formation and growth rates (Fig. 2a, Table 2). The growth rate of the strains 5D-*atp2* and 5D-*atpAGD* dropped by 35% and that of strain 5D-*atpAGD* (2A) by 26%. A decrease in biomass corresponds to a decrease in biocatalyst for ethanol production and, consequently, the reduced biomass directly influenced the volumetric ethanol productivity, which was cut down by 45% and 10% for strains 5D-*atp2* and 5D-*atpAGD*, respectively, but only by 1% for 5D-*atpAGD* (2A). Clearly, while yields and specific productivities will be less affected, the relative changes in the volumetric productivities will depend on the total runtime (i.e., the amount of substrate used) of the respective cultivation. For longer cultivations, it can be expected that the volumetric productivity improves faster for the control strain due to the faster accumulation of biomass.

### Effect of ATPase expression on growth-decoupled (nitrogen-starved) ethanol production

The reduced volumetric productivity, observed despite improved product yield and partially improved specific productivities, is a well-documented effect of enforced ATP wasting as more product is made at the expense of biomass [11, 17, 18, 37]. Similar as in [17], we hypothesized that this trade-off can be overcome if growth and production are separated. This was tested in a growth-decoupled (nitrogen-starved) approach in which the strains were inoculated with a higher starting OD_600_ of ca. 2.5 (vs. ca. 0.2 used in the growth-coupled approach) in a minimal medium lacking the nitrogen source. The strains were then incubated at 30 °C and biomass, ethanol production as well as glucose uptake was monitored. It is important to note that yeast cells typically continue to grow for a while even upon nitrogen starvation. Here, protein degradation pathways and proteasome play a major role in nitrogen recycling via extensive degradation of RNA and proteins [38–41]. To minimize biomass formation under nitrogen starvation, the cells grown on minimal medium (containing nitrogen) were first pre-cultured in a medium without nitrogen (to deplete cellular nitrogen reservoirs) before transfer to the final nitrogen-starved medium (see “Materials and methods”).

**Fig. 3:**
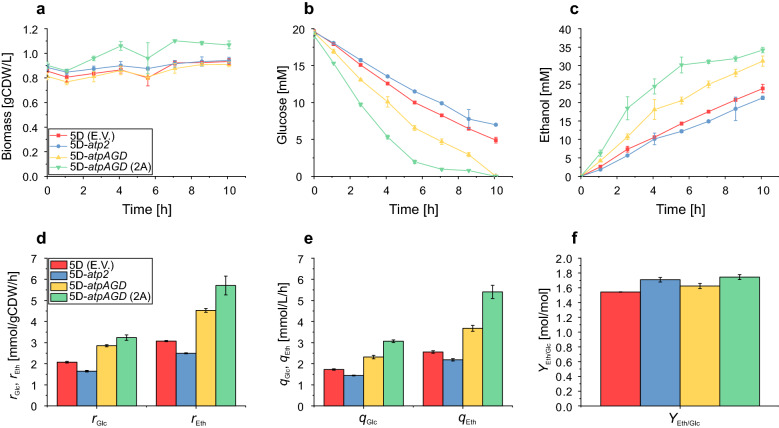
Biomass formation (**a**), glucose consumption (**b**), ethanol production (**c**), specific glucose uptake rate/ethanol productivity (**d**), volumetric glucose uptake rate/ethanol productivity after 5.5 h (**e**) and ethanol yield (**f**) of the different strains under growth-decoupled (nitrogen-starved) conditions. Specific and volumetric rate values are reported for the first 5.5 h of cultivation. Yields are reported for the entire cultivation period (0–10 h). The data presented are from triplicates and the error bars represent standard deviations

With this protocol, we observe that all strains show nearly constant biomass concentrations over the course of the cultivation (10 h), although a slight increase can be seen for 5D-*atpAGD* (2A) during the first 4 h (Fig. 3). Under these growth-decoupled conditions, the specific glucose uptake rate and specific ethanol production rate of the two strains 5D-*atpAGD* and 5D-*atpAGD* (2A) expressing the three ATPase subunit genes from *E. coli* are significantly higher than in the control strain. Consequently, as hypothesized, the volumetric productivity of these strains shows a relative improvement of more than 44% and 111%, respectively, thus overcoming the negative impact of reduced biomass on volumetric productivity. The increased specific and volumetric ethanol productivities can again be explained by the higher ATP turnover as indicated by the estimated non-growth-associated ATP maintenance demand (Table 3). In contrast, and in agreement with the growth-coupled experiments, expression of the β-subunit from *T. reesei* was not beneficial and even reduced specific as well as volumetric productivity. At the same time, the ethanol yield was again higher in all ATPase-expressing strains compared to the control strain.

**Table 3:** Growth rate, glucose uptake and production of ethanol, glycerol and acetate of the constructed *S. cerevisiae* strains under growth-decoupled (nitrogen-starved) cultivation

	**5D (E.V.)**	5D-*atp2*	5D-*atpAGD*	5D-*atpAGD* (2A)
µ [h^−1^]	~ 0	~ 0	~ 0	~ 0
*r*_Glc_ [mmol/gCDW/h]	2.07 ± 0.05	1.65 ± 0.05 (*p* = 0.0004)	2.85 ± 0.06 (*p* < 0.0001)	3.24 ± 0.16 (*p* = 0.0003)
*q*_Glc_ [mmol/L/h]	1.73 ± 0.04	1.44 ± 0.02 (*p* = 0.0004)	2.32 ± 0.10 (*p* = 0.0007)	3.07 ± 0.08 (*p* < 0.0001)
*r*_Eth_ [mmol/gCDW/h]	3.07 ± 0.03	2.50 ± 0.03 (*p* < 0.0001)	4.53 ± 0.11 (*p* < 0.0001)	5.71 ± 0.55 (*p* = 0.0011)
*q*_Eth_ [mmol/L/h]	2.56 ± 0.07	2.19 ± 0.07 (*p* = 0.0033)	3.68 ± 0.17 (*p* = 0.0005)	5.40 ± 0.38 (*p* = 0.0002)
*Y*_Eth/Glc_ [mol/mol]	1.54 ± 0.00	1.71 ± 0.04 (*p* = 0.0017)	1.62 ± 0.04 (*p* = 0.0294)	1.75 ± 0.04 (*p* = 0.0011)
*r*_Gly_ [mmol/gCDW/h]	0.386 ± 0.017	0.331 ± 0.005 (*p* = 0.0058)	0.444 ± 0.009 (*p* = 0.0060)	0.490 ± 0.011 (*p* = 0.0008)
*Y*_Gly/Glc_ [mol/mol]	0.172 ± 0.003	0.192 ± 0.005 (*p* = 0.0039)	0.138 ± 0.005 (*p* = 0.0006)	0.144 ± 0.002 (*p* = 0.0002)
*r*_Ac_ [mmol/gCDW/h]	0.0503 ± 0.0041	0.0413 ± 0.0108 (*p* = 0.2506)	0.0666 ± 0.0109 (*p* = 0.0713)	0.0779 ± 0.0058 (*p* = 0.0025)
*Y*_Ac/Glc_ [mol/mol]	0.0198 ± 0.0014	0.0228 ± 0.0041 (*p* = 0.2931)	0.0230 ± 0.0038 (*p* = 0.2309)	0.0249 ± 0.0028 (*p* = 0.0443)
*r*_ATP-NGAM_ [mmol/gCDW/h]	2.88	2.33	4.31	5.47

Specific and volumetric rate values are reported for the first 5.5 h of cultivation. Yields are reported for the entire period of cultivation (0–10 h). The data are from triplicate experiments and the error ranges represent standard deviations. *p* values for a two-sample *t* test are given with respect to the control strain 5D (E.V.) indicating whether a significant change has occurred

*Y*_BM/Glc_, biomass yield; *r*_Glc_, specific glucose uptake rate; *q*_Glc_*,* volumetric glucose uptake rate; *r*_Eth_, specific ethanol production rate; *q*_Eth_, volumetric ethanol productivity; *Y*_Eth/Glc_, ethanol yield; *r*_Gly_, specific glycerol production rate; *Y*_Gly/Glc_, glycerol yield; *r*_Ac_, specific acetate production rate*Y*_Ac/Glc_, acetate yield; *r*_ATP-NGAM_, non-growth-associated ATP maintenance demand (estimated from flux balance calculations)

To sum up, our experimental results provide a conclusive evidence of activity of *E. coli* F_1_-ATPase in *S. cerevisiae* and its beneficial impact on ethanol production under anaerobic growth-coupled (especially on ethanol yield) and growth-decoupled (especially on productivity) conditions.

## Discussion

Enforced ATP wasting has been recognized as a promising metabolic engineering strategy to enhance the microbial production of metabolites [11, 17, 18, 42–44] and it also appears to be suitable to improve production of ethanol by yeast. Along this line, we extended and complemented previous studies by (1) constructing yeast strains that overproduce ATPase (subunits) yielding sufficient ATPase activity, and (2) using those strains to comprehensively study the effect of ATPase expression in *S. cerevisiae* on growth, substrate utilization as well as ethanol production in defined (minimal) medium.

ATPase activity in *S. cerevisiae* was enabled via the expression of F_1_-ATPase genes from either *T. reesei* (β subunit as originally proposed in [21]) in strain 5D-*atp2* or from *E. coli* (αβγ subunits as previously used in [15, 17, 18]). The subunit genes of the *E. coli* ATPase were placed either under the control of single promoters in strain 5D-*atpAGD* or joined via viral 2A sequences to enable polycistronic expression in strain 5D-*atpAGD* (2A). Genomic integration of single copies of these ATPase genes did not lead to significant ATPase activity for any of the variants tested. One possible reason is the low copy number; another explanation could be that the chosen X2 locus [35] was not suitable to achieve sufficiently high expression rates. In contrast, significant enzymatic ATPase activities could be measured for plasmid-based expression of *E. coli*’s F_1_-ATPase genes in the two strains 5D-*atpAGD* and 5D-*atpAGD* (2A), both with comparable activity levels. We could not find such a conclusive proof of a functional ATPase activity in previous studies. For example, whereas the expression of a ribosome-associated molecular chaperone with ATP-hydrolyzing activity improved ethanol production on glucose and galactose as substrate, the enzymatic activity of ATP hydrolysis was reported to be reduced by almost 20%; similarly the expression of ATP-degrading apyrase enzyme showed a 20% improvement in enzymatic activity of ATP hydrolysis but did not result in improvement of ethanol production by the engineered strains [14].

In our experiments, neither genomic integration nor episomal expression of the genes of the β subunit of the ATPase from *T. reesei* led to enhanced ATPase activity or to a phenotype that would be consistent with increased ATPase activity. In the original work suggesting the heterologous production of the β subunit of *T. reesei’s* F_1_-ATPase to increase ATP drain in *S. cerevisiae* [21], ATPase activities were not measured and only a minor increase in ethanol production was reported. Together with our experimental data on metabolic fluxes and yields, which indicate a typical phenotype of a strain overexpressing a (metabolically inactive) protein (reduced growth rate, only slightly reduced biomass yield), we, therefore, postulate that the β subunit is indeed produced in strain 5D-*atp2* but does not have functional ATPase activity.

As one key result, our study proves a beneficial effect of expressing *E. coli*’s F_1_-ATPase on ethanol production by engineered *S. cerevisiae* strains. Under growth-coupled conditions, the ethanol yield significantly improved for strains expressing ATPase because a larger fraction of the substrate is used for synthesizing ATP (coupled to ethanol synthesis). However, for the same reason, enforced ATP wasting results in a reduced growth and biomass formation. This implies that less biocatalyst is available for ethanol production compared to the control strain which has up to 26% more biomass for ethanol production (Table 2) leading to higher volumetric productivities by the control strain. This drawback, however, can be overcome by two-stage fermentations where growth and production are decoupled. As a proof-of-principle for such an approach, we showed that growth-decoupled (nitrogen-starved) production, where the biomass of both the control and ATPase-expressing strains are comparable, the yield, specific productivity as well as the volumetric productivity of the ATPase-expressing strains were considerably improved (Fig. 3, Table 3). The positive impact of ATP wasting during growth-decoupled production phase was also discussed in a recent theoretical study [37]. Interestingly, even though there are several studies on ATP wasting and its impact on improved metabolite production in both prokaryotic as well as eukaryotic hosts, data on application of ATP-wasting techniques on growth-arrested cells in literature are scarce; only recently the potential of ATP wasting to improve product synthesis during growth-decoupled conditions was studied in *E. coli* [17].

Our study thus demonstrates that enforced ATP wasting in yeast can either be used to increase ethanol yield (especially under growth-coupled conditions) or for improving the volumetric productivity of ethanol synthesis with high yield under growth-decoupled conditions where the negative effect of ATPase activity on growth is not relevant. This trade-off between yield and volumetric productivity should be considered specifically for each application. A two-stage cultivation with separated growth and production phase comes at the cost of producing biomass in the beginning, which is overcome in the production phase in terms of higher productivity. The use of immobilized cells or their reuse via retention can further help to minimize these drawbacks.

Expression of ATPase usually also affects substrate uptake rate. In *E. coli*, expression of the F_1_-ATPase genes resulted in an increase in specific glucose uptake rate of more than 18% and 130% under growth-coupled and growth-decoupled conditions, respectively, which contributes to the observed increased specific productivity in *E. coli* [17]. In the two yeast strains with increased ATPase activity, an increase in specific glucose uptake rates of 37–56% was also observed under growth-decoupled conditions (Table 3), whereas no significant change or even a decrease could be seen under growth-coupled conditions. Thus, compared to *E. coli*, it seems that *S. cerevisiae* might have a reduced capacity (or regulatory hurdles) to counteract the loss of ATP by increasing its glycolytic flux for enhanced ATP synthesis during growth, similar as it has been reported for *Lactococcus lactis* [15]. Generally, from the two strains expressing *E. coli*’s F_1_-ATPase genes, the strain with polycistronic expression (5D-*atpAGD* (2A)) consistently showed slightly higher ATPase activity and superior ethanol production performance compared to strain 5D-*atpAGD* with single promotors for each gene. This indicates that the polycistronic expression may lead to a higher level of active F_1_-ATPase.

## Conclusions

We have successfully constructed yeast strains overexpressing ATPase genes and showed that the resulting increased ATPase activity leads to beneficial effects on ethanol yield or volumetric ethanol production, depending on the chosen process type. The plasmids generated in this work offer a simple and practical way to enforce ATP wasting in *S. cerevisiae* and can be easily employed in other applications. Even though this study aims to provide a proof-of-principle for the application of enforced ATP wasting in yeast, the use of auxotrophic markers makes these plasmids also attractive for industrial use owing to their stability in yeast cells [45].

In future work, it will be interesting to study effects of ATP wasting in *S. cerevisiae* with substrates other than glucose (such as maltose, sucrose) or to improve the production of other target metabolites that are coupled to energy generation. Those studies will help to uncover the full potential of enforced ATP wasting as a valuable tool for metabolic engineering of yeast cells.

## Materials and methods

### Strain construction and plasmids

Expression cassettes of the three ATPase variants used in this study were synthesized by GeneArt (Thermo Fisher Scientific) and were directly cloned into the corresponding target-vector. The Easyclone marker-less intergrative vector set for *S. cerevisiae* using CRISPR-Cas9 was employed for genomic integration of the ATPase encoding genes at X2 locus as described in the user manual [35]. The plasmids expressing Cas9 and helper gRNA were removed by growing the cells on non-selective medium. Successful integration at X2 locus was confirmed via colony PCR using primers according to the protocol [35]. For episomal expression, pRSII326 [36] was used and purchased from Addgene.

Viral 2A peptide sequences were used to enable polycistronic expression of the *E. coli* ATPase in *S. cerevisiae*. In a recent study comparing more than 20 different 2A peptide sequences, the 2A peptides from Equine rhinitis B virus (ERBV-1) and Porcine teschovirus-1 (PTV) showed the best cleavage efficiency in *S. cerevisiae* [30] and were chosen for use in this study. The ERBV-1 peptide sequence was used between the *atpA* and *atpG* whereas the PTV peptide sequence was used between the *atpG* and *atpD*.

The sequences of the ATPase constructs are provided in Additional file 1 and all strains and plasmids used in this study are listed in Table 4.

**Table 4:** Strains and plasmids used in this study

	Description	Reference
*Strain*		
7D	*S. cerevisiae* CEN.PK113-7D. MATa; URA3; TRP1; LEU2; HIS3; MAL2-8c; SUC2	Euroscarf
7D-*atp2*	7D with genomic integration of the *T. reesei* ATPase β subunit gene *atp2* (pTEF1-*atp2*-tCYC1) at X2 locus	This study
7D-*atpAGD* (2A)	7D with genomic integration of the *E. coli* ATPase α_,_ β and γ subunit genes *atpAGD* joined via viral 2A peptides at X2 locus	This study
5D	*S. cerevisiae* CEN.PK113-5D. MATa; ura3-52; TRP1; LEU2; HIS3; MAL2-8c; SUC2	Euroscarf
5D (E.V.)	5D transformed with the empty vector pRSII326	This study
5D-*atp2*	5D transformed with pRSII326-*atp2*	This study
5D-*atpAGD*	5D transformed with pRSII326-*atpAGD*	This study
5D-*atpAGD* (2A)	5D transformed with pRSII326-*atpAGD* (2A)	This study
*Plasmids*		
pCfB2312	Cas9 Expression Vector	[35]
pCfB2899	EasyClone-MarkerFree Integrative Vector for insertion into Chromosome X: 194944...195980 (X2 locus)	[35]
pCfB3020	EasyClone-MarkerFree gRNA Helper Vector for insertion into X2 locus	[35]
pCfB2899-*atp2*	pCfB2899 carrying the *T. reesei* ATPase β subunit gene *atp2* (pTEF1-*atp2*-tCYC1)	This study
pCfB2899-*atpAGD* (2A)	pCfB2899 carrying the *E. coli* ATPase α_,_ β and γ subunit genes *atpAGD* joined with viral 2A peptide sequences under the control of TEF1 promoter	This study
pRSII326	Shuttle vector with URA3 marker and 2µ ORI-STB. Addgene ID: 35469	[36]
pRSII326-*atp2*	pRSII326 with *T. reesei* ATPase β subunit gene *atp2* (pTEF1-*atp2*-tCYC1)	This study
pRSII326-*atpAGD*	pRSII326 with *E. coli* ATPase α subunit gene *atpA* (pTPI-*atpA*-tCPS1), subunit β gene *atpD* (pTEF1-*atpD*-tPRM9) and γ subunit gene *atpG* (pGPM1-*atpG*-tHIS5)	This study
pRSII326-*atpAGD* (2A)	pRSII326 with *E. coli* ATPase α_,_ β and γ subunit genes *atpAGD* joined with viral 2A peptide sequences under the control of TEF1 promoter	This study

### Cultivation conditions

Overnight cultures of the yeast strains were routinely grown aerobically at 30 °C (shaker with shaking amplitude of 50 mm and 190 rpm) in SD-URA (6.7 g/L yeast nitrogen base without amino acids, 1.92 g/L URA dropout medium). The overnight culture was centrifuged and washed using water before inoculating the final medium with a given OD_600_. The experiments to analyze the effect of ATPase expression were performed in mineral salt medium [46] with the following composition: 5 g/L (NH_4_)_2_SO_4_, 3 g/L KH_2_PO_4_, and 0.5 g/L MgSO_4_·7H_2_O. Trace elements and vitamins were added to the medium and their 1000 × stock was prepared with following compositions. 1000 × trace elements stock: 15 g/L Na_2_EDTA, 4.5 g/L ZnSO_4_·7H_2_O, 1 g/L MnCl_2_·2H_2_O, 0.3 g/L CoCl_2_·6H_2_O, 0.3 g/L CuSO_4_·5H_2_O, 0.4 g/L Na_2_MoO_4_·2H_2_O, 4.5 g/L CaCl_2_·2H_2_O, 3 g/L FeSO_4_·7H_2_O, 1 g/L H_3_BO_3_ and 0.1 g/L KI. 1000 × vitamins stock: 0.05 g/L d-biotin, 1 g/L Ca-d-pantothenate, 1 g/L nicotinic acid, 25 g/L myo-inositol, 1 g/L thiamine hydroxychloride, 1 g/L pyridoxine hydrochloride, and 0.2 g/L *p*-aminobenzoic acid. For experiments under anaerobic conditions, the mineral salt medium was additionally supplemented with ergosterol (7 mg/L) and Tween 80 (1:1200) as described previously [47]. For experiments on growth-decoupled (nitrogen-starved) ethanol production, (NH_4_)_2_SO_4_ was left out of the medium to suppress biomass formation. Furthermore, cultures were preadapted to anaerobic conditions and nitrogen-starved to empty intracellular nitrogen reservoirs before actual measurements started. Therefore, aerobic overnight cultures in SD-URA (2% glucose) were centrifuged, washed with water, and inoculated into minimal medium with nitrogen (2% glucose). After 6–8 h of anaerobic cultivation at 30 °C, cells were centrifuged, washed with water, and inoculated into minimal medium without nitrogen (2% glucose). After growth arrest (10–11 h anaerobic cultivation at 30 °C) cells were centrifuged, washed with water, and inoculated into minimal medium without nitrogen (0.4% glucose) for measurement of glucose consumption and ethanol production for 10 h of anaerobic cultivation at 30 °C.

For anaerobic cultivation, 50-ml cultivation tubes (Falcon) with screw cap were used, filled with 30–50 mL cultivation medium and incubated at 30 °C with stirring in an anaerobic chamber (don whitley scientific, United Kingdom) with an oxygen-free atmosphere composed of 10% H_2_, 10% CO_2_ and 80% N_2_.

### Analytical methods

ATPase activity of the cell lysates was measured using the ATPase Activity Assay Kit (Colorimetric) from BioVision (#K417). Pellets were made from strains cultivated anaerobically in 10 mL mineral salt medium and were stored at -80 °C until further use. Cell disruption was done via chemical lysis with CellLytic Y (Sigma) supplemented with 10 mM DTT and EDTA-free protease inhibitor cocktail (Roche) according to the manufacturers protocol. Lysates were centrifuged at 10,000×*g* for 10 min at 4 °C. Supernatants were transferred to Eppendorf tubes (or 1.5-mL reaction tubes) and further treated according to the manufacturer’s protocol. The ATPase activity was normalized to the total protein content in the lysate which was determined via measuring the absorbance at 280 nm on a NanoDrop 1000 spectrometer (peqlab Biotechnologie GmbH).

An Agilent 1100 series HPLC system equipped with a REZEX-ROA column (phenomenex, 00H-0138-K0, 300 × 7.8 mm) was used to quantify glucose, ethanol, glycerol and acetate in sample supernatants using 4 mM H_2_SO_4_ as liquid phase. Filtered samples were separated at 65 °C and 0.5 mL/min for 35 min with detection using a refractive index detector. Data were analyzed using the Chromeleon (Thermo Scientific) software package.

The specific rates for growth-coupled experiments were measured using the following formula:

$$r = \mu \left( {c_{M,e} {-}c_{M,s} } \right) \, / \, \left( {c_{X,e} {-}c_{X,s} } \right) \quad \left[ {{\text{mmol}}/{\text{gCDW}}/{\text{h}}} \right],$$

where *µ* is the growth rate, *c*_*M,e*_ and *c*_*M,s*_ represent the end and *s*tart concentrations of the respective metabolite M (mmol/L glucose, ethanol, glycerol, or acetate) and *c*_*X,e*_ and *c*_*X,s*_ represent the end and *s*tart concentrations of the biomass (gCDW/L). In growth-decoupled experiments, the biomass concentration remained nearly constant and, therefore, the following formula was employed for calculating the specific rates:

$$r = \, \left( {c_{M,e} {-}c_{M,s} } \right)/X_{{{\text{Av}}}} /\Delta t \quad \left[ {{\text{mmol}}/{\text{gCDW}}/{\text{h}}} \right],$$

where *X*_Av_ is the average biomass concentration (gCDW/L), and Δ*t* = *t*_*e*_*-t*_*s*_ the length of the time period (difference of end and start time).

For the volumetric productivities (in both growth-coupled and growth-decoupled cultivations), we used the formula:

$$q = \, \left( {c_{M,e} {-}c_{M,s} } \right) / \Delta t \quad \left[ {{\text{mmol}}/{\text{L}}/{\text{h}}} \right].$$

Yields were determined by plotting the amount of synthesized product (ethanol, glycerol, acetate, biomass) against the consumed glucose for every measurement time point and computing the slope of the linear regression, which then represents the respective yield.

The respective time periods used for the calculations of rates, productivities and yields are mentioned in the text.

## Supplementary information

**Additional file 1.** DNA sequence of genes and regulatory elements used in this study.

## Data Availability

The authors declare that all data supporting the findings of this study are available within the paper and its Supplementary Information files or are available from the corresponding author on request.
