# Supplementary material for: ATPase-based implementation of enforced ATP wasting in Saccharomyces cerevisiae for improved ethanol production
Source: Biotechnol Biofuels. 2020 Nov 9;13:185. doi: 10.1186/s13068-020-01822-9 (PMC7654063; doi:10.1186/s13068-020-01822-9)
Supplement: Supplementary file 1 — Additional file 1. DNA sequence of genes and regulatory elements used in this study. [file 13068_2020_1822_MOESM1_ESM.docx]

**Additional file 1**

**ATPase-based implementation of enforced ATP wasting in
*Saccharomyces cerevisiae* for improved ethanol production**

Ahmed Zahoor^1^, Katrin Messerschmidt^1^, Simon Boecker^1^, Steffen Klamt^1^

^1^Analysis and Redesign of Biological Networks, Max Planck Institute for Dynamics of Complex Technical Systems, Magdeburg, Germany

**DNA sequence of genes and regulatory elements used in this study**

1. pTEF1

TCCCCCACACACCATAGCTTCAAAATGTTTCTACTCCTTTTTTACTCTTCCAGATTTTCTCGGACTCCGCGCATCGCCGTACCACTTCAAAACACCCAAGCACAGCATACTAAATTTCCCCTCTTTCTTCCTCTAGGGTGTCGTTAATTACCCGTACTAAAGGTTTGGAAAAGAAAAAAGAGACCGCCTCGTTTCTTTTTCTTCGTCGAAAAAGGCAATAAAAATTTTTATCACGTTTCTTTTTCTTGAAAATTTTTTTTTTTGATTTTTTTCTCTTTCGATGACCTCCCATTGATATTTAAGTTAATAAACGGTCTTCAATTTCTCAAGTTTCAGTTTCATTTTTCTTGTTCTATTACAACTTTTTTTACTTCTTGCTCATTAGAAAGAAAGCATAGCAATCTAATCTAAGTTTTAATTACAAA

2. pTPI

TATATCTAGGAACCCATCAGGTTGGTGGAAGATTACCCGTTCTAAGACTTTTCAGCTTCCTCTATTGATGTTACACCTGGACACCCCTTTTCTGGCATCCAGTTTTTAATCTTCAGTGGCATGTGAGATTCTCCGAAATTAATTAAAGCAATCACACAATTCTCTCGGATACCACCTCGGTTGAAACTGACAGGTGGTTTGTTACGCATGCTAATGCAAAGGAGCCTATATACCTTTGGCTCGGCTGCTGTAACAGGGAATATAAAGGGCAGCATAATTTAGGAGTTTAGTGAACTTGCAACATTTACTATTTTCCCTTCTTACGTAAATATTTTTCTTTTTAATTCTAAATCAATCTTTTTCAATTTTTTGTTTGTATTCTTTTCTTGCTTAAATCTATAACTACAAAAAACACATACATAAACTAAAA

3. pGPM1

TAGTCGTGCAATGTATGACTTTAAGATTTGTGAGCAGGAAGAAAAGGGAGAATCTTCTAACGATAAACCCTTGAAAAACTGGGTAGACTACGCTATGTTGAGTTGCTACGCAGGCTGCACAATTACACGAGAATGCTCCCGCCTAGGATTTAAGGCTAAGGGACGTGCAATGCAGACGACAGATCTAAATGACCGTGTCGGTGAAGTGTTCGCCAAACTTTTCGGTTAACACATGCAGTGATGCACGCGCGATGGTGCTAAGTTACATATATATATATATATATATATATATATATATATAGCCATAGTGATGTCTAAGTAACCTTTATGGTATATTTCTTAATGTGGAAAGATACTAGCGCGCGCACCCACACACAAGCTTCGTCTTTTCTTGAAGAAAAGAGGAAGCTCGCTAAATGGGATTCCACTTTCCGTTCCCTGCCAGCTGATGGAAAAAGGTTAGTGGAACGATGAAGAATAAAAAGAGAGATCCACTGAGGTGAAATTTCAGCTGACAGCGAGTTTCATGATCGTGATGAACAATGGTAACGAGTTGTGGCTGTTGCCAGGGAGGGTGGTTCTCAACTTTTAATGTATGGCCAAATCGCTACTTGGGTTTGTTATATAACAAAGAAGAAATAATGAACTGATTCTCTTCCTCCTTCTTGTCCTTTCTTAATTCTGTTGTAATTACCTTCCTTTGTAATTTTTTTTGTAATTATTCTTCTTAATAATCCAAACAAACACACATATTACAATA

4. tCYC1

ATCCGCTCTAACCGAAAAGGAAGGAGTTAGACAACCTGAAGTCTAGGTCCCTATTTATTTTTTTATAGTTATGTTAGTATTAAGAACGTTATTTATATTTCAAATTTTTCTTTTTTTTCTGTACAGACGCGTGTACGCATGTAACATTATACTGAAAACCTTGCTTGAGAAGGTTTTGGGACGCTCGAAG

5. tCPS1

GCGCAATGATTGAATAGTCAAAGATTTTTTTTTTTTAATTTTTTTTTTTTAATTTTTTTTTTTTTTCATAGAACTTTTTATTTAAATAAATCACGTCTATATATGTATCAGTATAACGTAAAAAAAAAAACACCGTCAGTTAAACAAAACATAAATAAAAAAAAAAAGAAGTGTCAAATCAAGTGTCAAAT

6. tPRM9

CAGAAGACGGGAGACACTAGCACACAACTTTACCAGGCAAGGTATTTGACGCTAGCATGTGTCCAATTCAGTGTCATTTATGATTTTTTGTAGTAGGATATAAATATATACAGCGCTCCAAATAGTGCGGTTGCCCCAAAAACACCACGGAACCTCATCTGTTCTCGTACTTTGTTGTGACAAAGTAGCTCACTGCCTTATTATCACATTTTCATTATGCAACGCTTCGGAAAATACGATGTTGAAAAT

7. tHIS5

ATAGATTAATTTAAACAGTATATGTACAGTTTTATATATATATATATATATATATACATATATAAAGAAACCTGTGCGTTTTTTGTATTTTCAAATACATTTAGTTTTGGCGCCACTTCTATAAAAGGTCTCATGATATTGTTAC

8. *atp2* (*T. reesei*)

ATGTTCAAGAGCGGCGTTTCGTCCCTCGCCAGGGCTGCCCGCCCATCAATTACCGCTCGACGAGCTATCCGACCAGCCTTCCCTCGAACCCCCCTCGCGAGGCTTGCCAGCACCCAGAGCGTCGGAGATGGCAAGATCCACCAGGTCATTGGTGCCGTCGTCGACGTCAAGTTCGACACCGCCAAGCTGCCTCCTATCCTGAACGCCCTGGAGACCACCAACAACAACCAGAAGCTGGTCCTCGAGGTCGCTCAACACTTGGGCGAGAATGTCGTTCGCTGCATTGCCATGGACGGTACCGAGGGTCTCGTCCGTGGTTCCAAGGCCACTGACACCGGTGCCCCCATCACCATCCCCGTCGGCCCTGCCACTCTCGGTCGTATCCTGAACGTCACTGGTGACCCCATTGACGAGCGTGGCCCTGTCAAGACCGACAAGTTCCTGCCCATCCACGCCGACCCCCCGGCTTTCACTGACCAGTCCACCTCTGCCGAGATTCTGGTCACCGGTATCAAGGTCGTCGATCTGCTCGCTCCCTACGCTCGTGGTGGAAAGATTGGTCTGTTCGGTGGTGCCGGTGTCGGCAAGACCGTCTTCATCCAGGAGCTCATCAACAACATCGCCAAGGCCCACGGTGGTTACTCCGTCTTCACTGGTGTCGGTGAGCGTACCCGTGAGGGTAACGATCTGTACCACGAAATGCAGGAGACCTCCGTCATTCAGCTTGATGGCGAGTCCAAGGTCGCTCTGGTCTTCGGTCAGATGAACGAGCCCCCGGGAGCCCGTGCTCGTGTCGCCCTGACTGGTCTGACCATTGCCGAGTACTTCCGTGACCAGGAGGGCCAGGATGTCCTGCTCTTCATCGACAACATTTTCCGATTCACCCAGGCCGGTTCTGAGGTGTCTGCCCTGCTTGGTCGTATCCCCTCTGCCGTCGGTTACCAGCCCACCCTCGCCGTCGACATGGGTGGTATGCAGGAGCGAATCACCACCACCAAGAAGGGTTCCATTACCTCCGTCCAGGCCGTCTACGTCCCTGCTGACGATTTGACCGATCCTGCCCCCGCCACCACCTTCGCTCACTTGGACGCCACCACTGTCTTGTCTCGTGGTATCTCCGAGCTGGGTATCTACCCCGCCGTCGACCCTCTTGACTCAACGTCCCGTATGCTTGACCCCCGTATCGTCGGCCAGGAGCACTACCAGACCGCCACCCGCGTCCAGCAGATCCTCCAGGAGTACAAGGGTCTGCAAGATATCATTGCCATTCTGGGTATGGACGAACTGTCCGAAGCCGACAAGCTTACCGTCGAGCGTGCCCGAAAGATCCAGCGTTTCCTCAGCCAGCCCTTCACCGTCGCCCAGGTCTTCACTGGTATCGAGGGCAAGCTCGTCGACCTCAAGGACACCATTGCCTCCTTCAAGGCCATCCTCAACGGCGAGGGTGACAGCCTGCCCGAGGCTGCCTTCTACATGGTTGGCGACCTGGCTTCCGCCAAGGCCAAGGGTGAGAAGATTCTGGCGGAGTTGGAGAAGAACTAA

9. *atp1* (*E. coli*)

ATGCAACTGAATTCCACCGAAATCAGCGAACTGATCAAGCAGCGCATTGCTCAGTTCAATGTTGTGAGTGAAGCTCACAACGAAGGTACTATTGTTTCTGTAAGTGACGGTGTTATCCGCATTCACGGCCTGGCCGATTGTATGCAGGGTGAAATGATCTCCCTGCCGGGTAACCGTTACGCTATCGCACTGAACCTCGAGCGCGACTCTGTAGGTGCGGTTGTTATGGGTCCGTACGCTGACCTTGCCGAAGGCATGAAAGTTAAGTGTACTGGCCGTATCCTGGAAGTTCCGGTTGGCCGTGGCCTGCTGGGCCGTGTGGTTAACACTCTGGGTGCACCAATCGACGGTAAAGGTCCGCTGGATCACGACGGCTTCTCTGCTGTAGAAGCAATCGCTCCGGGCGTTATCGAACGTCAGTCCGTAGATCAGCCGGTACAGACCGGTTATAAAGCCGTTGACTCCATGATCCCAATCGGTCGTGGTCAGCGTGAATTGATCATCGGTGACCGTCAGACAGGTAAAACCGCACTGGCTATCGATGCCATCATCAACCAGCGCGATTCCGGTATCAAATGTATCTATGTCGCTATCGGCCAGAAAGCGTCCACCATTTCTAACGTGGTACGTAAACTGGAAGAGCACGGCGCACTGGCTAACACCATCGTTGTGGTAGCAACCGCGTCTGAATCCGCTGCACTGCAATACCTGGCACCGTATGCCGGTTGCGCAATGGGCGAATACTTCCGTGACCGCGGTGAAGATGCGCTGATCATTTACGATGACCTGTCTAAACAGGCTGTTGCTTACCGTCAGATCTCCCTGCTGCTCCGTCGTCCGCCAGGACGTGAAGCATTCCCGGGCGACGTTTTCTACCTCCACTCTCGTCTGCTGGAGCGTGCTGCACGTGTTAACGCCGAATACGTTGAAGCCTTCACCAAAGGTGAAGTGAAAGGGAAAACCGGTTCTCTGACCGCACTGCCGATTATCGAAACTCAGGCGGGTGACGTTTCTGCGTTCGTTCCGACCAACGTAATCTCCATTACCGATGGTCAGATCTTCCTGGAAACCAACCTGTTCAACGCCGGTATTCGTCCTGCGGTTAACCCGGGTATTTCCGTATCCCGTGTTGGTGGTGCAGCACAGACCAAGATCATGAAAAAACTGTCCGGTGGTATCCGTACCGCTCTGGCACAGTATCGTGAACTGGCAGCGTTCTCTCAGTTTGCATCCGACCTTGACGATGCAACACGTAAGCAGCTTGACCACGGTCAGAAAGTGACCGAACTGCTGAAACAGAAACAGTATGCGCCGATGTCCGTTGCGCAGCAGTCTCTGGTTCTGTTCGCAGCAGAACGTGGTTACCTGGCGGATGTTGAACTGTCGAAAATTGGCAGCTTCGAAGCCGCTCTGCTGGCTTACGTCGACCGTGATCACGCTCCGTTGATGCAAGAGATCAACCAGACCGGTGGCTACAACGACGAAATCGAAGGCAAGCTGAAAGGCATCCTCGATTCCTTCAAAGCAACCCAATCCTGGTAA

10. *atp2* (*E. coli*)

ATGGCTACTGGAAAGATTGTCCAGGTAATCGGCGCCGTAGTTGACGTCGAATTCCCTCAGGATGCCGTACCGCGCGTGTACGATGCTCTTGAGGTGCAAAATGGTAATGAGCGTCTGGTGCTGGAAGTTCAGCAGCAGCTCGGCGGCGGTATCGTACGTACCATCGCAATGGGTTCCTCCGACGGTCTGCGTCGCGGTCTGGATGTAAAAGACCTCGAACACCCGATTGAAGTCCCGGTAGGTAAAGCGACTCTGGGCCGTATCATGAACGTACTGGGTGAACCGGTCGACATGAAAGGCGAGATCGGTGAAGAAGAGCGTTGGGCGATTCACCGCGCAGCACCTTCCTACGAAGAGCTGTCAAACTCTCAGGAACTGCTGGAAACCGGTATCAAAGTTATCGACCTGATGTGTCCGTTCGCTAAGGGCGGTAAAGTTGGTCTGTTCGGTGGTGCGGGTGTAGGTAAAACCGTAAACATGATGGAGCTCATTCGTAACATCGCGATCGAGCACTCCGGTTACTCTGTGTTTGCGGGCGTAGGTGAACGTACTCGTGAGGGTAACGACTTCTACCACGAAATGACCGACTCCAACGTTATCGACAAAGTATCCCTGGTGTATGGCCAGATGAACGAGCCGCCGGGAAACCGTCTGCGCGTTGCTCTGACCGGTCTGACCATGGCTGAGAAATTCCGTGACGAAGGTCGTGACGTTCTGCTGTTCGTTGACAACATCTATCGTTACACCCTGGCCGGTACGGAAGTATCCGCACTGCTGGGCCGTATGCCTTCAGCGGTAGGTTATCAGCCGACCCTGGCGGAAGAGATGGGCGTTCTGCAGGAACGTATCACCTCCACCAAAACTGGTTCTATCACCTCCGTACAGGCAGTATACGTACCTGCGGATGACTTGACTGACCCGTCTCCGGCAACCACCTTTGCGCACCTTGACGCAACCGTGGTACTGAGCCGTCAGATCGCGTCTCTGGGTATCTACCCGGCCGTTGACCCGCTGGACTCCACCAGCCGTCAGCTGGACCCGCTGGTGGTTGGTCAGGAACACTACGACACCGCGCGTGGCGTTCAGTCCATCCTGCAACGTTATCAGGAACTGAAAGACATCATCGCCATCCTGGGTATGGATGAACTGTCTGAAGAAGACAAACTGGTGGTAGCGCGTGCTCGTAAGATCCAGCGCTTCCTGTCCCAGCCGTTCTTCGTGGCAGAAGTATTCACCGGTTCTCCGGGTAAATACGTCTCCCTGAAAGACACCATCCGTGGCTTTAAAGGCATCATGGAAGGCGAATACGATCACCTGCCGGAGCAGGCGTTCTACATGGTCGGTTCCATCGAAGAAGCTGTGGAAAAAGCCAAAAAACTTTAA

11. *atp3* (*E. coli*)

ATGGCCGGCGCAAAAGAGATACGTAGTAAGATCGCAAGCGTCCAGAACACGCAAAAGATCACTAAAGCGATGGAGATGGTCGCCGCTTCCAAAATGCGTAAATCGCAGGATCGCATGGCGGCCAGCCGTCCTTATGCAGAAACCATGCGCAAAGTGATTGGTCACCTTGCACACGGTAATCTGGAATATAAGCACCCTTACCTGGAAGACCGCGACGTTAAACGCGTGGGCTACCTGGTGGTGTCGACCGACCGTGGTTTGTGCGGTGGTTTGAACATTAACCTGTTCAAAAAACTGCTGGCGGAAATGAAGACCTGGACCGACAAAGGCGTTCAATGCGACCTCGCAATGATCGGCTCGAAAGGCGTGTCGTTCTTCAACTCCGTGGGCGGCAATGTTGTTGCCCAGGTCACCGGCATGGGGGATAACCCTTCCCTGTCCGAACTGATCGGTCCGGTAAAAGTGATGTTGCAGGCCTACGACGAAGGCCGTCTGGACAAGCTTTACATTGTCAGCAACAAATTTATTAACACCATGTCTCAGGTTCCGACCATCAGCCAGCTGCTGCCGTTACCGGCATCAGATGATGATGATCTGAAACATAAATCCTGGGATTACCTGTACGAACCCGATCCGAAGGCGTTGCTGGATACCCTGCTGCGTCGTTATGTCGAATCTCAGGTTTATCAGGGCGTGGTTGAAAACCTGGCCAGCGAGCAGGCCGCCCGTATGGTGGCGATGAAAGCCGCGACCGACAATGGCGGCAGCCTGATTAAAGAGCTGCAGTTGGTATACAACAAAGCTCGTCAGGCCAGCATTACTCAGGAACTCACCGAGATCGTCTCGGGGGCCGCCGCGGTTTAA
